# A bipolar functionality of Q/N-rich proteins: Lsm4 amyloid causes clearance of yeast prions

**DOI:** 10.1002/mbo3.83

**Published:** 2013-03-20

**Authors:** Keita Oishi, Hiroshi Kurahashi, Chan-Gi Pack, Yasushi Sako, Yoshikazu Nakamura

**Affiliations:** 1 Department of Basic Medical Sciences, Institute of Medical Science, University of Tokyo4-6-1 Shirokanedai, Minato-ku, Tokyo, 108-8639, Japan; 2 Cellular Informatics Laboratory, RIKEN Advanced Science InstituteWako-shi, Saitama, 351-0198, Japan

**Keywords:** Amyloid, Lsm4, Pin^+^ factor, Q/N-rich protein, yeast prion.

## Abstract

Prions are epigenetic modifiers that cause partially loss-of-function phenotypes of the proteins in *Saccharomyces cerevisiae*. The molecular chaperone network that supports prion propagation in the cell has seen a great progress in the last decade. However, the cellular machinery to activate or deactivate the prion states remains an enigma, largely due to insufficient knowledge of prion-regulating factors. Here, we report that overexpression of a [*PSI*^+^]-inducible Q/N-rich protein, Lsm4, eliminates the three major prions [*PSI*^+^], [*URE3*], and [*RNQ*^+^]. Subcloning analysis revealed that the Q/N-rich region of Lsm4 is responsible for the prion loss. Lsm4 formed an amyloid in vivo, which seemed to play a crucial role in the prion elimination. Fluorescence correlation spectroscopy analysis revealed that in the course of the Lsm4-driven [*PSI*^+^] elimination, the [*PSI*^+^] aggregates undergo a size increase, which ultimately results in the formation of conspicuous foci in otherwise [*psi*^−^]-like mother cells. We also found that the antiprion activity is a general property of [*PSI*^+^]-inducible factors. These data provoked a novel “unified” model that explains both prion induction and elimination by a single scheme.

## Introduction

Prions are proteinaceous infectious particles lacking nucleic acid (Prusiner 1982). Prion proteins can generally adopt two distinct conformations, normal and prion, the latter of which is prone to forming amyloid structures (Pan et al. 1993). The mammalian prion protein PrP is pathogenic, and is believed to be the causative agent for a series of transmissible spongiform encephalopathies (Prusiner 2001). In the last two decades, a number of prions were identified in *Saccharomyces cerevisiae*. Three major yeast prions are [*PSI*^+^], [*URE3*], and [*RNQ*^+^], which are caused by amyloidogenesis of translation termination factor Sup35, nitrogen metabolism regulator Ure2, and functionally uncharacterized protein Rnq1, respectively (Wickner 1994; Wickner et al. 1995; Sondheimer and Lindquist 2000). Prions in yeast are not necessarily pathogenic, but rather act as an epigenetic switch to yield a partially loss-of-function phenotype of the respective protein function (reviewed in Liebman and Chernoff 2012). For example, the [*PSI*^+^] prion causes frequent translational readthrough at the nonsense codons (Cox 1965), with numerous readthrough protein products synthesized that might contribute to enhanced cellular adaptability to deleterious environments.

Many proteins are involved in supporting the prion biology in yeast. In particular, the chaperones network of Hsp40s, Hsp70s, and Hsp104 has been extensively studied, and now is known to play crucial roles in the regulation of prion propagation in yeast (reviewed in Sweeny and Shorter 2008). However, the molecular bases by which cells undergo the de novo prion induction and prion curing are still poorly understood, mainly due to little knowledge of cellular factors that influence on the prion switching. To date, overexpression of several Pin^+^ ([*PSI*^+^]-inducible) factors such as Rnq1 and Lsm4 is known to enhance the frequency of the de novo [*PSI*^+^] prion induction (Derkatch et al. 2001), while overexpression of late endosome-associated proteins Btn2 (Kryndushkin et al.,^2008^), G protein γ-subunit mimic Gpg1 (Ishiwata et al., 2009), and a series of Rnq1 mutants (Kurahashi et al. 2008; Shibata et al. 2009) eliminates [*PSI*^+^].

A member of the Lsm protein family, Lsm4, exploits its N-terminal region (Kambach et al., 1999) to play an important role in mRNA processing and degradation in the nucleus and cytoplasm, respectively (Mayes et al. 1999; Tharun et al. 2000). Having long been uncharacterized, its C-terminal amyloid-prone glutamine(Q)/asparagine(N)-rich region was recently revealed to play a role in the assembly of P-bodies (Decker et al. 2007). Also, as a Pin^+^ protein, Lsm4 is known to facilitate the de novo [*PSI*^+^] appearance (Derkatch et al. 2001) and its Q/N-rich region has been shown to form amyloids (Alberti et al. 2009), suggesting the involvement of its Q/N-rich region in the prion biology. In this study, we found that overexpression of Lsm4 causes the elimination of preexisting prions in yeast, and this antiprion activity may be generally shared among the proteins bearing Q/N-rich regions.

## Experimental Procedures

### Strains

*Saccharomyces cerevisiae* strains used in this study are as follows: NPK50 ([*PSI*^+^] [*rnq*^−^] *MATa ade1-14 leu2-3,112 ura3-52 his3Δ200 trp1-289*) (74-D694), NPK200 ([*psi*^−^] [*RNQ*^+^] isogenic with NPK50) (OT60 [Bailleul et al. 1999]), NPK51 ([*psi*^−^] [*rnq*^−^] *MATa ade1-14 leu2-3,112 ura3-52 his3Δ200 trp1-289*) (Kurahashi et al. 2008), NPK301 ([*ure-o*] [*rnq*^−^] *MATa PD-ADE2 his3 leu2 trp1 kar1 PD-CAN1*) (BY242 [Brachmann et al. 2005]), NPK302 ([*URE3*] [*rnq*^−^] isogenic with NPK301), ND32 (*ura3Δ0 Δlsm4*^*91–187*^ derivative of NPK302) (this study), NPK377 ([*ure-o*] [*rnq*^−^] *hsp104::LEU2* derivative of NPK301), ND21 ([*PSI*^+^] [*rnq*^−^] *MATa ade1-14 leu2 ura3 hi3 trp1 sup35::SUP35-GFP*), ND20 ([*psi*^−^] [*rnq*^−^] isogenic with ND21) (ND21 and ND20 are derivative of G74-D694) (Kawai-Noma et al. 2009), NPK294 ([*PSI*^+^] [*RNQ*^+^] *MATa ade1-14 leu2-3,112 ura3-52 his3Δ200 trp1-289*) (Kurahashi et al. 2008), and NA124 ([*PSI*^+^] [*RNQ*^+^] *sup35::HA-SUP35 pdr5::KanMX* derivative of NPK294).

### Plasmids and PCR primers

Plasmids used are pRS400 series vectors (Stratagene, La Jolla, CA), in which promoters are placed at the *Sac*I-*Bam*HI cassette and the *CYC1* terminator is placed at the *Xho*I-*Kpn*I cassette. In this study, plasmids are denoted by pRS400YYYp-XXX where XXX is the gene to be expressed under the control of the promoter of YYY. When Lsm4, Rnq1, or Sup35NM is tagged with GFP or mRFP (monomeric RFP) at its C-terminus, the *LSM4*, *RNQ1*, *SUP35NM* gene is placed at the *Bam*HI-*Sal*I cassette, and the *GFP* or *mRFP* gene is placed at the *Sal*I-*Xho*I cassette. For the construction of *LSM4*-expressing plasmid, the coding sequence of *LSM4* was amplified by PCR using P1 and P2 primers for the *Bam*HI-*Xho*I insertion. For the construction of pRS413LSM4p-LSM4-mRFP, the LSM4p-LSM4 region was directly amplified without the *Bam*HI site insertion using P3 and P4 for the *Sac*I-*Sal*I insertion, and mRFP was amplified using P5 and P6 for the *Sal*I-*Xho*I insertion. For the construction of plasmids expressing Lsm4-truncated mutants, the *LSM4* open reading frame (ORF) was used as the template and amplified by PCR using P1 and P7 for *LSM4*^*1–90*^ to be inserted into the *Bam*HI-*Xho*I site, P1 and P8 for *LSM4*^*1–110*^, P1 and P9 for *LSM4*^*1–130*^, P1 and P10 for *LSM4*^*1–150*^, P1 and P11 for *LSM4*^*1–170*^, and P12 and P2 for *LSM4*^*91–187*^. For the N-terminally HA-tagged proteins, the coding sequence of the HA-tag was placed at the end of the *GPD* promoter cassette by PCR amplification using P13. For constructing pET15b plasmids carrying *LSM4* or one of its truncated mutants in the *Nde*I-*Xho*I cassette, P14 was used as the forward primer containing the *Nde*I site and either P2, P7, P8, P9, P10, or P11 was used as the reverse primer. For the construction of plasmids carrying Pin^+^ factors in the *Bam*HI-*Xho*I cassette, the ORF of each gene was amplified by PCR using a pair of P15 and P16 for *NEW1*; P17 and P18 for *STE18*; P19 and P20 for *SWI1*; P21 and P22 for *PIN2*; P23 and P24 for *URE2*; P25 and P26 for *PIN4*; P27 and P28 for *CYC8*; P29 and P30 for *URE2*; P31 (for *Bgl*II digestion) and P32 for *PIN3*; P33 and P34 for *YCK1*; P35 (for *BgI*II digestion) and P36 for *NUP116*. For constructing a plasmid carrying *RNQ1* in the *Bam*HI-*Xho*I cassette, *RNQ1* ORF was obtained by digesting one of the plasmids from our previous study (Kurahashi et al. 2008). pVTG12 is a generous gift from Wickner and colleague, and has been described elsewhere (Edskes et al. 1999). The primer sequences are listed in Table S1.

### Protein analysis

Sodium dodecyl sulfate polyacrylamide gel electrophoresis (SDS-PAGE) and semi-denaturing detergent agarose gel electrophoresis (SDD-AGE) were carried out as described previously (Kryndushkin et al. 2003; Liebman et al. 2006; Kurahashi and Nakamura 2007). The immunoblot experiments were performed using anti-Sup35C antibody (Nakayashiki et al. 2001), anti-Rnq1 antibody (Kurahashi and Nakamura 2007), anti-Lsm4 antibody (prepared in this study), anti-HA antibody (3F10, Roche Applied Science, Penzberg, Upper Bavaria, Germany), anti-FLAG antibody (M2, Sigma-Aldrich, St. Louis, MO), anti-Hsp104 antibody (Affinity Bioreagents, Rockford, IL), and anti-Pgk1 antibody (Molecular Probes, Eugene, OR).

### Immunoprecipitation

Experiments were performed essentially as described previously (Kurahashi et al. 2008), except that magnetic beads were used in this study. Cells were broken by vortexing for 45 sec twice at 4°C in NP-40 lysis buffer (150 mmol/L NaCl, 1.0% NP-40, 50 mmol/L Tris pH 8.0, adequate amount of complete protease inhibitor cocktail [Roche Applied Science, Penzberg, Upper Bavaria, Germany]) with glass beads. Crude lysates were cleaned by a 5600 rpm spin for 10 min in an Eppendorf benchtop centrifuge. Sixty microliters of lysates were first incubated with adequate amount of antibodies for 3.5 h, and subsequently incubated with 5 μL of Dynabeads Protein G beads for 3 h. Precipitates were washed with NP-40 lysis buffer three times before resuspension in gel loading buffer for SDS-PAGE and western blot analysis.

### Fluorescence microscopy

Fluorescence microscopy was performed using a MetaMorph apparatus (Universal Imaging Corporation, Marlow, Buckinghamshire, U.K.) attached to an IX71 microscope (Olympus, Tokyo, Japan). For the assessment of Lsm4-prion colocalization, fluorescence microscopy was performed using a confocal microscope A1 (Nikon, Tokyo, Japan).

### Thermotolerance

Experiments were performed as described previously (Tkach and Glover 2004). Briefly, cells in log phase in YPD at 30°C were preincubated at 37°C for 60 min to induce Hsp104 with the heat-shock response and then transferred to a 50°C water bath for 20 min. Aliquots of cells were transferred to ice immediately and survival of cells in these samples was determined by titration on YPD media.

### Thioflavin T assay

Protein was purified in a denaturing condition as described previously (Crist et al. 2003). Purified protein was buffer exchanged by dialysis with a buffer with 8 mol/L of urea and 20 mmol/L of Tris at pH 7.4. In vitro Thioflavin T assay was then performed as prescribed previously (Chernoff et al. 2002).

### Fluorescence correlation spectroscopy

All the FCS measurements were taken at 25°C on LSM510 confocal microscope combined with a ConfoCor 2 (Zeiss, Oberkochen, Baden-Württemberg, Germany), as described in previous studies (Kawai-Noma et al. 2006, 2009; Pack et al. 2006; Kurahashi et al. 2011).

## Results

### Propagation of [*PSI*^+^] is inhibited upon overexpression of *LSM4*

**Figure 1:**
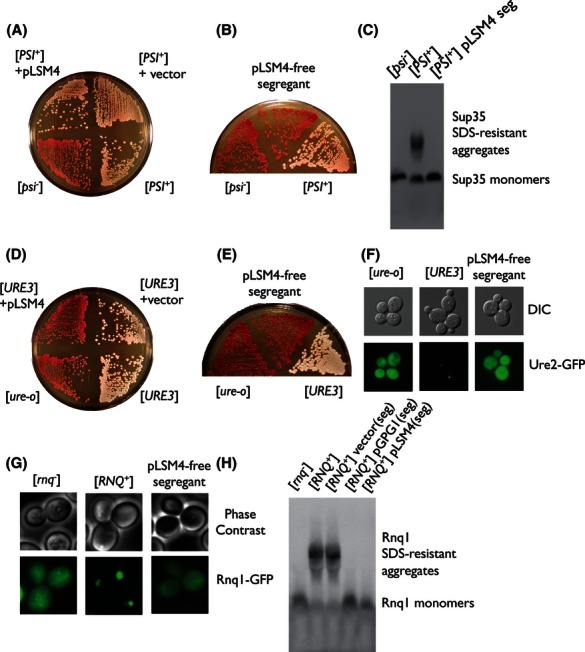
Overexpression of *LSM4* cures [*PSI*^+^], [*URE3*], and [*RNQ*^+^]. (A) [*PSI*^+^] elimination by pLSM4. [*PSI*^+^][*rnq*^−^] strain NPK50 was transformed with pRS425GPDp-LSM4 (denoting a multicopy *LEU2*^+^ plasmid expressing the *LSM4* gene under the control of the *GPD* promoter) or an empty vector pRS425. Transformants were grown and selected on SC-Leu, and regrown on YPD for 3 days for the red/white colony color assay. NPK50 and NPK51 were used for the [*PSI*^+^] and [*psi*^−^] controls, respectively. White and red represent the prion and prion-less colonies, respectively. (B) Confirmation of the [*PSI*^+^] loss in pLSM4-free segregants. The red colonies obtained in (A) were spread on YPD until the plasmid is spontaneously lost. The isolated segregants retained its red color after the plasmid loss. (C) Absence of [*PSI*^+^] aggregates confirmed by SDD-AGE. The pLSM4-free segregants in (B) were cultured to log phase in YPD, and exposed to SDD-AGE using anti-Sup35C antibody. The high-molecular-weight SDS-stable aggregates of Sup35, a hallmark of [*PSI*^+^], disappeared in the segregants (denoted as “seg”). (D) [*URE3*] elimination by pLSM4. [*URE3*] [*ure-o*] strain NPK302 was transformed with pRS425GPDp-LSM4 or an empty vector, and exposed to the colony color assay. The [*URE3*] and [*ure-o*] controls are NPK302 and NPK301, respectively. (E) pLSM4-free segregants exhibiting red color on YPD. (F) The [*ure-o*]-like fluorescent pattern of Ure2N-GFP in the pLSM4-free segregants. The segregants obtained in (B) were transformed with pVTG12, which expresses the N-domain of *URE2* fused to GFP under the authentic *URE2* promoter. Transformants were selected on SC-Leu plate, cultured to log phase, and subjected to fluorescence microscopy. (G) The [*rnq*^−^]-like fluorescent pattern of Rnq1-GFP in the pLSM4-free segregants. [*RNQ*^+^][*psi*^−^] cells (NPK200) were cotransformed with pRS415GAL1p-RNQ1-GFP (a single-copy *LEU2*^+^ plasmid expressing *RNQ1-GFP* under the *GAL1* promoter) and either pRS423GPDp-LSM4, pRS423GPDp-GPG1 (for the [*rnq*^−^] control), or an empty vector (for the [*RNQ*^+^] control). Transformants were directly cultured in SC-Leu-His liquid for at least 30 generations, and subsequently cultured in SC-Leu to drop pLSM4. After the transformants carrying pRNQ1-GFP but lacking pLSM4 were selected, 20 colonies were subjected to the fluorescence microscopy for each. (H) Absence of [*RNQ*^+^] aggregates confirmed by SDD-AGE. NPK200 was transformed with pLSM4, pGPG1, or an empty vector (the same plasmids as in [A]). Transformants were directly cultured in SC-His for more than 30 generations, and spread on YPD plates to drop the plasmid. Segregants (denoted as “seg”) were then cultured to log phase and subjected to the SDD-AGE experiment using anti-Rnq1 antibody.

The [*PSI*^+^] prion, caused by amyloid formation of the eRF3 peptide release factor Sup35, can be easily monitored in a test strain where *ADE1* gene on the adenine biosynthesis pathway harbors an opal premature termination codon (Nakayashiki et al. 2001). The *ade1-14* gene yields functional Ade1 protein only when the stop codon readthrough becomes frequent in the [*PSI*^+^] state. In [*psi*^−^] (i.e., non-[*PSI*^+^]), cells are incapable of the proper Ade1 biosynthesis, which causes the polymerization of a metabolic intermediate in the pathway and thereby results in a red shift of the colony color. Overexpression of *LSM4* under the strong promoter of *GPD* in a [*PSI*^+^] strain (NPK50) strain resulted in the white-to-red colony color conversion at a moderate (average = 54.0%, standard deviation = 14.7%) frequency (Fig. 1A). Those cells stayed red even after the *LSM4*-overexpressing plasmid was lost from the cells (Fig. 1B), which rules out the possibility that the red shift was brought about by unexpected alteration of adenine metabolism and which rather suggests that [*PSI*^+^] propagation was impaired by overexpression of *LSM4*.

The [*PSI*^+^] loss in the plasmid-free segregants was confirmed by the method of SDD-AGE, a protein electrophoresis to detect SDS-resistant aggregates as a distinctive characteristic associated with amyloids (Kryndushkin et al. 2003). Lysates prepared from the red segregant were subject to SDD-AGE experiment as described by Liebman et al. (2006). The SDD-AGE result showed that the SDS-stable aggregates of Sup35, which is present in the original [*PSI*^+^] cells, disappeared in the red cells where *LSM4* had been once overexpressed (Fig. 1C). This absence of Sup35 aggregates, along with the appearance of the [*psi*^−^]-like red colonies, strongly indicates that Lsm4 eliminated [*PSI*^+^] upon overexpression.

### Overexpression of *LSM4* cures two other major prions, [*URE3*] and [*RNQ*^+^]

We next asked whether *LSM4* overexpression could cure other major prions? [*URE3*], the longest-studied prion in *S. cerevisiae*, is the prion form of the nitrogen catabolism regulator Ure2. Like [*PSI*^+^], a colorimetric [*URE3*] reporter system has been established (Brachmann et al. 2005). In this system, the *ADE2* gene, another adenine marker, is placed under the chromosomal *DAL5* promoter. Ure2 inhibits the transcription factor Gln3, thus repressing the downstream *DAL5* expression. In the presence of [*URE3*], Ure2 proteins are recruited to the [*URE3*] prion amyloids and allow the production of Ade2, while in [*ure-o*] (i.e., loss-of-[*URE3*]) Ade2 fails to be produced with the formation of the red polymer of a pathway intermediate, thus turning cells red. We transformed a [*URE3*] strain, NPK302, with the same *LSM4*-overexpressing plasmid as above, and observed the red conversion of the transformant colonies at a very high (average = 100%, standard deviation = 0%) frequency (Fig. 1D). Those red colonies retained the color after the plasmid was dropped (Fig. 1E).

We confirmed the elimination of [*URE3*] by fluorescence microscopy. The [*URE3*] prion is inherently vulnerable to adding a GFP tag to the Ure2 protein, which makes it difficult to use Ure2-GFP fusion proteins for a reporter system (Edskes et al. 1999). However, Wickner and colleagues overcame this hurdle by developing a reporter plasmid, pVTG12, which expresses Ure2N-GFP, a fusion protein of the N-terminal Gln/Asn-rich domain (also known as the “prion domain”) of Ure2 and a GFP tag, in the native expression level (Edskes et al. 1999). With this plasmid, we successfully observed that the pLSM4-free segregants obtained above exhibited diffuse localization of Ure2N-GFP similar to [*ure-o*] positive control cells, whereas [*URE3*] negative control cells exhibited punctate foci of Ure2N-GFP that represent the presence of the [*URE3*] prion aggregates (Fig. 1F). From these data, we concluded that Lsm4 inhibits the prion propagation of [*URE3*] upon overexpression.

We also tested if Lsm4 is capable of destabilizing the third major yeast prion [*RNQ*^*+*^]. [*RNQ*^*+*^] is a prion of Rnq1, a protein of no known function that was named after its amyloid-forming region “rich in N (asparagine) and Q (glutamine),” and is known to facilitate the de novo induction of the [*PSI*^+^] prion (Derkatch et al. 1997, 2001; Osherovich and Weissman 2001). Although intensive studies have been conducted for the [*RNQ*^*+*^] prion as a good example of prion–prion interactions, very few colony color-based reporter system remains to be developed largely due to lack of the knowledge of Rnq1's molecular function. True and colleagues have developed a color-based assay for [*RNQ*^+^] (Bardill and True 2009), but replacement of the non-Q/N-rich region of Rnq1 by that of Sup35 may alter the authentic behavior of [*RNQ*^+^]. Therefore, we chose to perform fluorescence microscopy and SDD-AGE analysis for assessment of the ability of Lsm4 to cure [*RNQ*^*+*^]. For the fluorescence microscopy experiment, Lsm4 was strongly expressed in [*RNQ*^*+*^] for >30 generations, pLSM4-free segregant cells were prepared, and the Rnq1-GFP markers were expressed in the segregants to monitor [*RNQ*^*+*^]/[*rnq*^−^] cell states. We observed that overexpression of Lsm4 caused [*RNQ*^+^] prion loss in 20% of tested colonies (Fig. 1G), whereas Gpg1, a G-protein γ subunit previously found by our group to strongly disturb multiple yeast prions (Ishiwata et al., 2009), caused [*RNQ*^+^] prion loss in 75% of tested colonies under the same experimental condition (data not shown). The anti-[*RNQ*^*+*^] activity of Lsm4 was verified by the SDD-AGE experiment (Fig. 1H).

Collectively, we discovered that Lsm4 is a novel general inhibitor of yeast prions. Note that no growth inhibition effect was observed in either [*PSI*^+^] or [*URE3*] cells transformed with the *LSM4* overexpressing plasmid, precluding the possibility that overproduced Lsm4 actually selected for nonprion cells (Fig. S1A and B). Hereafter, we mainly used [*URE3*], curing of which was the most sensitive to *LSM4* overexpression, for further investigation.

### The Q/N-rich region of Lsm4 is responsible for the prion-curing effect

Lsm4 is a member of the Lsm protein family highly conserved in a wide range of organisms from archea to humans and plays important roles both in nucleus and cytoplasm (reviewed in Tharun 2009). In the nucleus, Lsm4 along with Lsm2 to Lsm8 forms ring-like heteroheptameric complex to bind to and stabilize U6 snRNA, thus playing a significant role in mRNA splicing (Mayes et al. 1999). On the other hand, in the cytoplasm, Lsm4 forms a similar ring-like complex with Lsm1 to Lsm7, which is recruited to P-body, an intracellular supercomplex of mRNA degrading factors, and is thus involved in mRNA decay (Tharun et al. 2000). The two roles of Lsm proteins are both achieved via intermolecular interaction of their N-terminal “Sm motifs” (Kambach et al. 1999).

**Figure 2:**
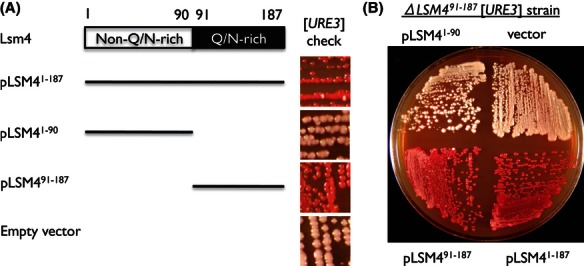
The Q/N-rich region of Lsm4 possesses antiprion activity. (A) The [*URE3*] loss by pLSM4^91–187^. [*URE3*][*rnq*^−^] strain NPK302 was transformed with pRS425GPDp-LSM4 (expressing full-length Lsm4), pRS425GPDp-LSM4^1–90^ (non-Q/N-rich region; residues 1–90), pRS425GPDp-LSM4^91–187^ (Q/N-rich region; residues 91–187), or an empty vector. Transformants were subjected to the colony color assay. (B) Independence of the curing process from mRNA processing/decay. Coding region of Lsm4's Q/N-rich region was deleted from NPK302 to establish a new strain (ND32) where Lsm4's Q/N-rich region is orthogonal to its non-Q/N-rich region. The experiment as in (A) was then conducted using the ND32 strain.

One unique primary-structural feature of the essential protein Lsm4 is the C-terminal region highly rich in glutamine and asparagine. The Q/N-rich region of Lsm4 apparently has no cellular function, except that it plays a role in the assembly of P-body units (Decker et al. 2007). To assess whether Lsm4 eliminates prion via its known mRNA-associated function or not, we overexpressed only its functional N-terminal region (residues 1–90; Lsm4^1–90^) or its C-terminal region (residues 91–187; Lsm4^91–187^) in [*URE3*] cells of NPK302. Intriguingly, [*URE3*] elimination was observed only in the cells overexpressing Lsm4^91–187^, but not in the cells overexpressing Lsm4^1–90^, which suggests that Lsm4's Q/N-rich region is involved in the elimination mechanism (Fig. 2A).

### The prion-curing mechanism is independent of endogenous Lsm4

A possibility still remains that the cellular functionality of Lsm4's N-terminal functional domain, which is crucial for its RNA maturation and decay functions, is affected by overexpression of its Q/N-rich region, which many yeast amyloidogenic proteins are known to possess in common. If Lsm4's Q/N-rich region forms amyloids and recruits chromosomally expressed Lsm4 molecule, the cell could undergo a shortage of Lsm4 proteins and a partial dysfunction in the RNA-related mechanisms, which then leads to a prion loss. To test whether the Q/N-rich region-driven prion elimination is dependent on or independent of known physiology of Lsm4, we constructed a [*URE3*] strain where Lsm4's Q/N-rich region is absent and is therefore orthogonal to the N-terminal functional region. We overexpressed *LSM4*^*91–187*^ in the *ΔLSM4*^*91–187*^ strain, and observed that [*URE3*] elimination did occur, indicating that Lsm4's N-terminal functional region is not involved in the curing mechanism (Fig. 2B).

### Lsm4 forms an amyloid upon overexpression

Q/N-rich regions are known to cause amyloid formation and are shared among all prion proteins in *S. cerevisiae*. A recent study by Lindquist and colleagues has demonstrated that 6xHis-tagged and EYFP-tagged Q/N-rich region fragments of Lsm4 exhibit amyloid-like properties in vitro and in vivo, respectively (Alberti et al. 2009). However, it remains to be fully proved that the full-length Lsm4 can form amyloid-like structure in vivo.

**Figure 3:**
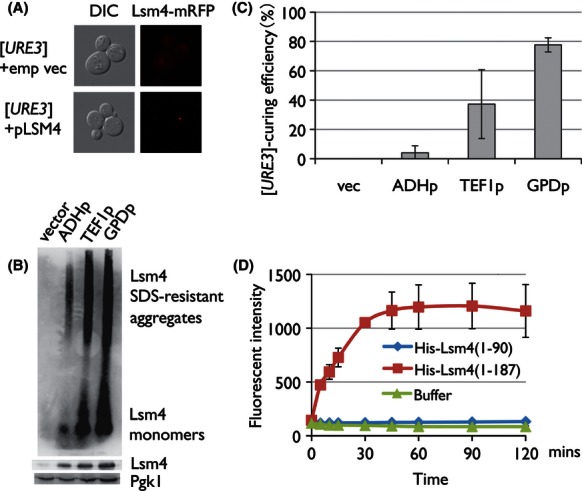
Lsm4 forms amyloid upon overexpression. (A) Emergence of Lsm4-mRFP foci on *LSM4* overexpression. NPK302 was cotransformed with pRS415LSM4p-LSM4-mRFP (expressing LSM4-mRFP under the authentic promoter) and pRS425GPDp-LSM4 or an empty vector. Transformants were selected on SC-Leu-His, cultured to log phase, and subjected to fluorescence microscopy. (B) SDS-stable aggregate formation of Lsm4 in vivo. NPK302 was transformed with a pRS415-based plasmid to express the *LSM4* gene under the control of the *GPD* (very strong), *TEF1* (strong), or *ADH* (moderate) promoter, or an empty vector. Transformants were selected and cultured on SC-Leu, and subjected to the SDD-AGE experiment. NPK302 itself possesses no SDS-stable aggregate (data not shown). (C) Dose dependency of Lsm4's prion curability. The transformants selected in (B) were subjected to the colony color assay. (D) Amyloid formation of Lsm4 in vitro. 6xHis-tagged Lsm4 and Lsm4^1–90^, which completely lacks the Q/N-rich region, were expressed in *Escherichia coli* using the pET15b expression system, and purified under a denaturing condition with 8 mol/L of urea, as previously described (Crist et al. 2003). The purified His-Lsm4 proteins were then subjected to the Thioflavin T-based amyloid formation assay. Lsm4 monomers were incubated in the Congo Red binding buffer (5 mmol/L potassium phosphate, pH 7.4, 150 mmol/L NaCl) with 20 μmol/L Thioflavin T using a revolution mixer (RVM-101, Asahi Techno Glass, Tokyo, Japan) in 30°C.

To assess its amyloidogenic nature, we expressed *LSM4-mRFP* under its native promoter with co-overexpression of full-length Lsm4. We observed Lsm4-mRFP punctate foci only when full-length Lsm4 is overexpressed (Fig. 3A). We also conducted an SDD-AGE experiment. We used single-copy plasmids to express full-length *LSM4* under a series of promoters of different strength (empty vector, *ADH*, *TEF1*, *GPD* promoters). In this SDD-AGE experiment, we followed the protocol developed by Kushnirov and colleagues (Kryndushkin et al. 2003), as Lsm4 monomers can hardly be detected in Liebman's protocol (Liebman et al. 2006) (data not shown). We observed that *LSM4* expression under any overexpressing promoters (*ADH*, *TEF1*, *GPD*) resulted in the formation of SDS-resistant high-molecular-weight structure in vivo (Fig. 3B). In contrast, when *LSM4* is normally expressed under its native promoter, Lsm4 was solely monomeric (i.e., non-SDS-resistant) and no high-molecular-weight structure was observed (Fig. 3B). The amyloidogenic nature of full-length Lsm4 was also confirmed in vitro via Thioflavin T-based assay (Fig. 3D). These data suggest that full-length Lsm4 can form amyloid-like structure in vivo and in vitro.

### Antiprion activity of Lsm4 is dosage dependent

Then what property of Lsm4's Q/N-rich region induces the prion elimination? To ask this question, we focused on the dosage dependency of the frequency at which overexpression of Lsm4 eliminates [*URE3*]. When Lsm4 was overexpressed under the moderate *ADH* promoter on a single-copy plasmid vector, [*URE3*] elimination was barely observed in the colony color assay. However, when Lsm4 was overexpressed under the stronger *TEF1* promoter and the even-stronger *GPD* promoter on the single-copy vector, [*URE3*] elimination was observed in approximately 40% and 80% of the cells tested, respectively (Fig. 3C). SDD-AGE analysis on these transformants revealed that the number of Lsm4's SDS-resistant aggregates and the size of the Lsm4 monomer pool both increased in a dosage-dependent manner (Fig. 3B). Together with the dosage-dependent curing frequency, this data suggest that antiprion activity of Lsm4's Q/N-rich region is driven by its amyloid-like polymerization or its monomer pool increase.

### Lsm4 amyloids may be responsible for the prion curing

**Figure 4:**
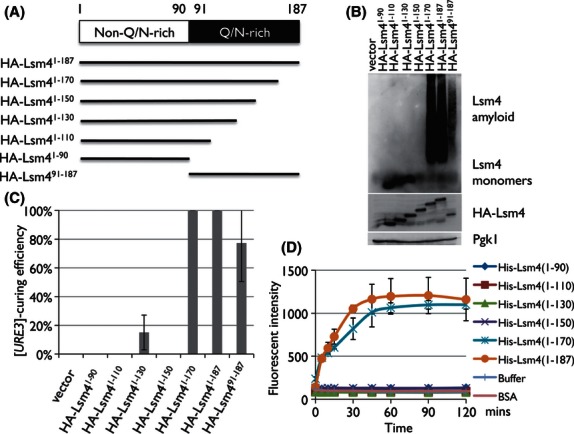
Amyloid formation of Lsm4 may be essential for the [*URE3*] elimination. (A) Schematic representation of the series of Lsm4 truncations used in this study. Each fragment was carried in the plasmid pRS425GPDp. (B, C) Positive correlation between Lsm4's amyloidogenicity and [*URE3*]-curability. [*URE3*][*rnq*] cells (NPK302) were transformed with a series of the pRS425-based plasmids where each of the C-terminally truncated mutants of *LSM4* is expressed under the *GPD* promoter. Transformants were subjected to the SDD-AGE analysis using the anti-HA antibody and the red/white colony color assay. (D) In vitro amyloidogenicity of each Lsm4 mutant. 6xHis-tagged Lsm4^1–90^, Lsm4^1–110^, Lsm4^1–130^, Lsm4^1–150^, Lsm4^1–170^_,_ and Lsm4^1–187^ (i.e., full-length Lsm4) were purified and subjected to the Thioflavin T-based amyloid formation assay, as described in Figure 3D.

The next questions are whether Lsm4 exploits its amyloid structure for prion curing and, if so, where the minimum functional region for both amyloid formation and prion curing is? To give insights into these questions, we constructed a series of multicopy plasmids expressing Q/N-rich region truncates of HA-tagged Lsm4 under the *GPD* promoter (HA-Lsm4^1–90^, HA-Lsm4^1–110^, HA-Lsm4^1–130^, HA-Lsm4^1–150^, HA-Lsm4^1–170^, HA-Lsm4^1–187^, HA-Lsm4^91–187^; see Fig. 4A). We then expressed the mutant series in [*URE3*] cells and investigated the in vivo amyloidogenicity and the [*URE3*]-curing frequency of each Lsm4 mutant by SDD-AGE and colony color assay, respectively (Fig. 4B and C). Surprisingly, the results indicated that there was a significant correlation between the amyloid-forming ability and the [*URE3*]-curing efficiency of each Lsm4 mutant. HA-Lsm4^1–187^ (full-length Lsm4) and HA-Lsm4^1–170^ as well as HA-Lsm4^91–187^ formed amyloid structures in vivo and exhibited very strong [*URE3*]-curing ability, while none of other mutants formed amyloid or showed the [*URE3*]-curing effect (Fig. 4B and C). One exception was HA-Lsm4^1–130^, which reproducibly exhibited a very weak curing effect, the reason of which is discussed in the Discussion section (Fig. 4B and C). Their amyloidogenicity was also assessed in vitro, using 6xHis-tagged Lsm4 mutants with the same truncations except Lsm4^91–187^. Consistent with the SDD-AGE result, the Thioflavin T-based in vitro assay indicated that His-Lsm4^1–170^ and His-Lsm4^1–187^ readily formed amyloid structures, while Lsm4^1–90^, Lsm4^1–110^, Lsm4^1–130^, Lsm4^1–150^ did not within 120 min tested (Fig. 4D). These data suggest that Lsm4 amyloid formation is responsible for the antiprion effect, and the amino acid region 150–170 may be important for Lsm4 amyloid formation.

### Size enlargement of [*PSI*^+^] prion amyloids occurs upon overexpression of *LSM4*

To investigate the effect of Lsm4 aggregates on the dynamics of prion amyloid particles, we conducted the fluorescence correlation spectroscopy (FCS) experiment to monitor the change in the diffusion coefficient of [*PSI*^+^] aggregates in a test cell (strain D21) in which GFP is integrated between the N and MC regions of the *SUP35* ORF. FCS allows us to determine the in vivo diffusion coefficient of Sup35-GFP by calculating the autocorrelation function in 1 femtoliter (10^−15^ L) detection volume, which then yields the size of the fluorescent [*PSI*^+^] particles.

**Figure 5:**
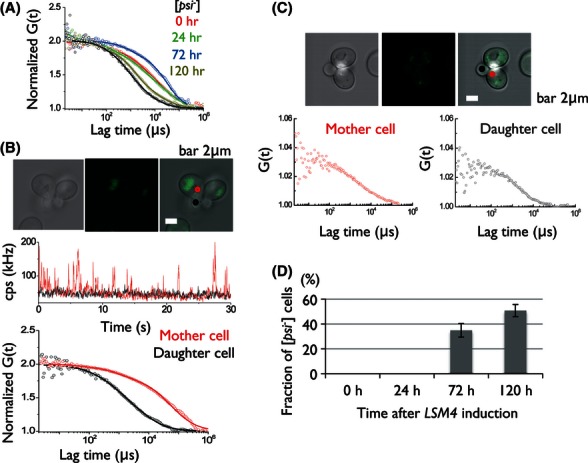
[*PSI*^+^] oligomers underwent size increase upon *LSM4* overexpression. The [*PSI*^+^][rnq^-^] *sup35::SUP35-GFP* strain (ND21) was transformed with pRS413GAL1p-LSM4 (expressing *LSM4* under the galactose-inducible *GAL1* promoter). Transformants were precultured to log phase in SSuc-His, induced the *LSM4* expression by galactose addition, and subjected to FCS at each time point. (A) Representative normalized autocorrelation functions of Sup35-GFP in mother cells from [*psi*^−^] cells (black), [*PSI*^+^] cells before *LSM4* induction (red), 24 (green), 72 (blue), and 120 h (brown) after *LSM4* induction. Fitting results by a two-component model are depicted by solid lines. (B) Mother–daughter asymmetry of the Sup35-GFP aggregation status observed at 72 h after *LSM4* induction. Top: The fluorescent image of the mother and daughter cell pair used for FCS measurement. Bar: 2 μm. Middle: Traces of average fluorescence intensities (counts per second: cps) for Sup35-GFP in a single [*PSI*^+^] cell pair of mother (red) and daughter (black). Bottom: Normalized fluorescence autocorrelation functions of the single [*PSI*^+^] cell pair of mother (red) and daughter (black), showing a clear contrast between the two. (C) Emergence of huge Sup35-GFP foci at 120 h after induction. Top: The fluorescent image of the cell pair of mother (red; foci present) and daughter (black) at 120 h. Bar: 2 μm. Bottom: Normalized fluorescence autocorrelation functions of the single cell pairs of the mother (red) and daughter (black), both showing a similar [*psi*^−^]-like pattern besides the presence of foci. (D) Fraction of [*psi*^−^] cells after the induction of *LSM4*.

We overexpressed *LSM4* under the galactose-inducible *GAL1* promoter in the [*PSI*^+^] cells. The FCS analysis showed that, in the first 24 h after induction, the average size of the Sup35-GFP [*PSI*^+^] particles did not change (Fig. 5A). Consistent to this, all cells were still [*PSI*^+^] according to the colony color test (Fig. 5D). However, after 72 h of induction, we observed a size enlargement of the diffusing [*PSI*^+^] particles in some mother–daughter cell pairs (Fig. 5A). Interestingly, such large aggregates were detected only in mother cells, while daughter cell exhibited Sup35-GFP particles diffusing as freely as the [*psi*^−^] control cells (Fig. 5B). After 120 h, a new, conspicuous type of cell pairs emerged where mother cells possess huge fluorescent foci, but mothers’ cytoplasm and the entire daughter cells exhibited the [*psi*^−^]-like diffusional behavior of Sup35-GFP (Fig. 5C). Consistently, colony color analysis showed that red colonies were observed at 72 h, and its ratio increased at 120 h (Fig. 5D). The FCS analysis indicates that the prion loss by *LSM4* overexpression was due to the failure of the prion transmission from mother to daughter cells, rather than the autonomous disappearance of prion aggregates in a single cell. Also, the data suggest that the curing mechanism involves an enlargement and number reduction of the [*PSI*^+^] aggregates, which tendency resembles the FCS data during the prion elimination by *rnq1Δ100* overexpression (Kurahashi et al. 2011) and GuHCl treatment (Kawai-Noma et al. 2010). The absence of [*PSI*^+^] particles and the presence of huge Sup35-GFP foci in mother cells after 120 h cannot be explained simply by the failure of [*PSI*^+^] particle transmission through the diffusion coefficient reduction because enlarged [*PSI*^+^] particles would simply continue to diffuse in the mother cell. Rather, this observation may raise a possibility that the enlarged prion particles were recruited to a certain compartment in a cell by some mechanism. Collectively, Lsm4-driven prion loss is caused by enlargement of the prion particle, followed by two different mechanisms: one is reduction of the diffusion coefficient of the prion particle in the cell, and the other is entrapment and transport of the enlarged prion particle to a certain compartment in the mother cell.

### The enlargement of prion particles may not be attributed to Hsp104 cellular functionality

If Lsm4 amyloid structures lead to the prion particle enlargement and prion loss, by what mechanism can this effect be achieved? One plausible scenario is the reduction of cellular functionality of Hsp104 by Lsm4 amyloid formation. Hsp104 is a general disaggregating chaperone that disentangles and resolubilizes a polypeptide from protein aggregates, thus indispensable for the thermotolerance of yeast cells (Sanchez and Lindquist 1990). This disentangling activity of Hsp104 is also essential for prion propagation (Parsell et al. 1994). For prion propagation, mother cells need to “cleave” their prion amyloids by Hsp104's activity and thereby generate new prion seeds for the transmission to the daughter cells. If Lsm4 amyloids bind to or inhibit Hsp104, the cellular availability of functional Hsp104 would be lowered and prion propagation will be inhibited with prion size enlargement.

To investigate this possibility, we first looked into the cellular abundance of Hsp104 after expressing *LSM4* under the strong *GPD* promoter on a multicopy plasmid in [*ure-o*] [*rnq*^−^] NPK302. Western blotting analysis using anti-Hsp104 antibody showed that there was no reduction or even a slight increase in the cellular Hsp104 abundance (Fig. S2A), suggesting that the prion particle enlargement itself cannot be attributed to alteration of the cellular abundance of Hsp104. Next, to address the possibility of reduction in Hsp104's cellular functionality, thermotolerance of the NPK301 transformant that overexpresses *LSM4* was evaluated. We cultured the transformant in SC-Leu liquid to the log phase at 30°C, induced heat-shock response by culturing it at 37°C for 1 h, and incubated the cells at 50°C at 20 min. The survival rate was determined by fivefold serial dilutions on YPD plates. As shown in Figure S2B, the heat-shock assay showed that there was no reduction of the thermotolerance of the *LSM4*-overexpressing transformant, which suggests that prion curing by Lsm4 may not be caused by reduced cellular functionality of Hsp104. However, heat-shock induction of Hsp104 expression complicates the proper interpretation of validity of the result. The absence of Hsp104 functionality reduction could be concluded under the assumption that Hsp104 abundances after heat shock are equal between the transformants with an empty vector and the *LSM4*-overexpressing plasmid. Further investigation needs to be conducted for dissecting the definite role of Hsp104 cellular functionality in the prion particle enlargement and curing.

### Lsm4 amyloid physically interacts with prion aggregates

**Figure 6:**
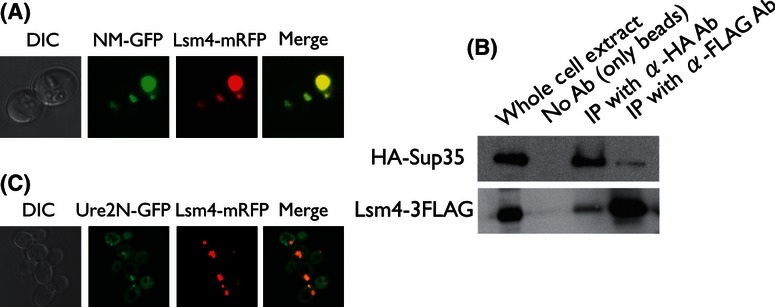
Physical interaction between Lsm4 and prions. (A) Colocalization of NM-GFP and Lsm4-mRFP. [*PSI*^+^] strain NPK294 was cotransformed with pRS424CUP1p-LSM4-mRFP and pRS415GAL1p-NM-GFP. Transformants were selected on SC-Leu-Trp plate, cultured in SGalSuc-Leu-Trp to mid-log phase to express NM-GFP, further cultured with 50 μmol/L CuSO_4_ for 3 h to express Lsm4-mRFP, and subjected to confocal microscopy (using Nikon A1) to visualize the two proteins’ intracellular localization. (B) Coimmunoprecipitation of HA-Sup35 and Lsm4-3FLAG. [*PSI*^+^] strain NA124 whose genomic *SUP35* ORF is replaced by *HA-SUP35* was transformed with pRS425GAL1p-LSM4-3FLAG. Transformants were selected on SC-Leu plate, precultured in SSuc-Leu liquid overnight, diluted and cultured in SGalSuc-Leu liquid to induce *LSM4-3FLAG* expression for 8 h, and subjected to the immunoprecipitation experiment as described in Experimental Procedures. (C) Colocalization of Ure2N-GFP and Lsm4-mRFP. [*URE3*] strain NPK302 was cotransformed with pRS424CUP1p-LSM4-mRFP and pVTG12 (*URE2N-GFP* placed under the authentic *URE2* promoter; *LEU2* marker; single copy). Transformants were selected on SC-Leu-Trp plate, precultured in SC-Leu-Trp liquid overnight, cultured in SC-Leu-Trp with 50 μmol/L CuSO_4_ to express *LSM4-mRFP* for 3 h, and subjected to confocal microscopy using Nikon A1.

If Hsp104 is unlikely to be the main cause of the prion enlargement and ensuing propagation failure, the next possible mechanism is that Lsm4 amyloid physically interacts with prion aggregates in cell, facilitating the increase in the prion aggregate size. To assess this possibility, we first conducted fluorescence microscopy to monitor the intracellular localizations of both Lsm4 and prion aggregates. We conditionally overexpressed GFP-tagged Sup35NM (NM-GFP) fragments in a [*PSI*^+^] strain under galactose-containing media and subsequently mRFP-tagged Lsm4 (Lsm4-mRFP) by adding CuSO_4_ (final concentration of 50 μmol/L). Using a confocal microscope, we observed a clear colocalization of Lsm4-mRFP and NM-GFP foci 3 h after Lsm4-mRFP induction (Fig. 6A). Nearly 100% of observed Lsm4-mRFP foci were colocalized with NM-GFP foci. To investigate the physical nature of the two proteins’ colocalization, we then conducted a coimmunoprecipitation experiment in a *sup35::HA-SUP35* [*PSI*^+^] strain (NA124) harboring a plasmid conditionally expressing 3FLAG-tagged Lsm4. Eight hours after Lsm4-3FLAG induction under a galactose-containing media, cells were harvested and subjected to coimmunoprecipitation using anti-HA antibody and anti-FLAG antibody. We then detected the coimmunoprecipitation of HA-Sup35 and Lsm4-3FLAG, implicating that the colocalization that we observed between Lsm4-mRFP and NM-GFP foci signified their physical interaction (Fig. 6B). We obtained a similar result for [*URE3*]; GFP-tagged N-terminal fragment of Ure2 (Ure2N-GFP) and mRFP-tagged Lsm4 (Lsm4-mRFP) showed a similar but somewhat less frequent colocalization (Fig. 6C). About 54% of observed Lsm4-mRFP foci were colocalized with Ure2N-GFP. Coimmunoprecipitation assay for Ure2 and Lsm4 could not be conducted due to the unavailability of an applicable anti-Ure2 antibody and the vulnerable nature of [*URE3*] against the expression of tagged Ure2 proteins.

### Many other Asn/Gln-rich proteins can also cure [*URE3*]

**Figure 7:**
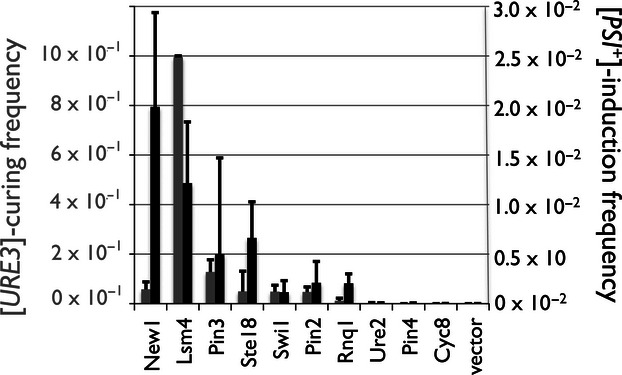
Many [*URE3*]-curable proteins tend to possess [*PSI*^+^]-inducibility. Gray: [*URE3*]-curing frequency of each Q/N-rich protein. NPK302 [*URE3*][*rnq*^−^] strain was transformed with a series of multicopy plasmids expressing one of the 10 Pin^+^ proteins under the *GPD* promoters (pRS425GPDp-XXX). Transformants were selected on SC-Leu plates and then subjected to the colony color assay. Curing frequency was semiquantitated by the fraction of the red (cured) colonies out of the total colonies on the YPD plate. At least three independent experiments were carried out. Bars denote the standard deviation. Black: [*PSI*^+^]-induction frequency of each Q/N-rich protein. NPK51 [*psi*^−^][*rnq*^−^] strain was cotransformed with a copper-dependent Sup35NM-expressing plasmid (pRS413CUP1p-SUP35NM) and each of the same Q/N-rich protein-expressing plasmids as above. Transformants were selected and cultured to log phase in SC-Leu-His, and then supplemented with CuSO_4_ to the final concentration of 50 μmol/L. Cultures were incubated at 30°C for 48 h. After brief wash, cells were spread on the [*PSI*^+^]-selective SC-Ade plates and nonselective SC plates for induction frequency quantitation. At least three independent experiments were carried out. Bars denote the standard deviation.

If the Q/N-rich region of Lsm4 is capable of eliminating preexisting prions, one may speculate that other proteins with Q/N-rich regions possess a similar ability to cure prions. We focused on the 11 [*PSI*^*+*^]-inducible Q/N-rich proteins that were identified by Liebman and colleagues (Derkatch et al. 2001; which include New1, Lsm4, Pin3, Ste18, Swi1, Pin2, Ure2, Pin4, Cyc8, Yck1, Nup116) and one classical [*PSI*^*+*^]-inducer, Rnq1. We overexpressed full-length sequences of those proteins under the strong promoter of *GPD* on a multicopy plasmid in [*URE3*] [*rnq*^−^] cells. Because severe growth inhibition was observed in the *YCK1*- and *NUP116*-overexpressing transformants, we stopped analyzing these two factors (data not shown). After transformant colonies appeared on SC selective media, each of the colonies was spread on a YPD medium and semiquantitated the [*URE3*]-curing frequency by calculating the percentage of red (cured) colonies. Interestingly, the colony colorimetric assay revealed that seven of the 10 factors tested exhibited the [*URE3*]-curing effect at the frequency higher than 1 × 10^−2^ (Fig. 7). These data reinforce the idea that Lsm4 Q/N-rich region is responsible for the prion elimination and, more importantly, suggest that the prion curability may be generally shared among many, but not all, Q/N-rich proteins.

### [*URE3*]-curable Q/N-rich proteins tend to possess [*PSI*^+^]-inducibility as well

In the above section, we found that Q/N-rich proteins are potential multicopy suppressors of prion propagation, and the curing frequencies vary among the Q/N-rich protein species. An important question here is what the difference is between the [*URE3*]-curable and non-[*URE3*]-curable Q/N-rich proteins? To find a key to answer this question, we focused on a well-known ability of Q/N-rich regions, namely [*PSI*^+^]-inducibility. To measure the efficiency of [*PSI*^+^] induction by each of the above Q/N-rich proteins, we cotransformed [*psi*^−^][*rnq*^−^] cells (NPK51) with a multicopy plasmid expressing each of the proteins under the constitutive *GPD* promoter and a single-copy plasmid expressing *SUP35NM* under the copper-inducible *CUP1* promoter. After culturing each of the transformants to the log phase at 30°C, we added CuSO_4_ to each culture to the final concentration of 50 μmol/L for the *SUP35NM* induction, and cultured them at 30°C for another 48 h. After brief washing, we spread the cells onto nonselective SC plates and selective SC-Ade plates simultaneously in order to calculate [*PSI*^+^] induction frequency. Surprisingly, the [*PSI*^+^]-inducing experiment showed that Q/N-rich proteins with [*URE3*]-curability tend to also possess [*PSI*^+^]-inducibility at the same time, suggesting that both processes may rely on a common physical property or cellular environment (Fig. 7). One deviation from this tendency is New1, which exhibited a very high frequency of [*PSI*^+^] induction but a low frequency of [*URE3*] elimination. The cause of this deviation remains unknown, but the homologous sequence repeat tracts present in both Sup35 and New1 may contribute to the efficient [*PSI*^+^] induction. Also, it remains unknown whether the [*PSI*^+^] induction was due to the de novo formation of the [*RNQ*^+^] prion, but it may be unlikely because some of the Q/N-rich proteins tested here have been shown to induce the de novo [*PSI*^+^] appearance in an *rnq1Δ* strain (Yang et al. 2013). Collectively, these data raise a possibility that both [*URE3*] curing and [*PSI*^+^] induction by Q/N-rich proteins like Lsm4 rely on their amyloid formation in the cell.

## Discussion

In this study, we have identified an Lsm protein family member, Lsm4, as a multicopy suppressor of prion propagation in yeast. The curing process occurs through the Q/N-rich C-terminal region, and is independent of the mRNA splicing or decay machinery. Lsm4 formed amyloid-like structures in vivo, and this amyloid formation of Lsm4 is probably essential for the impedance of prion propagation, as a strong correlation between amyloidogenicity and prion curability was observed in the C-terminally truncated mutants of Lsm4. One exception from the correlation was seen in Lsm4^1–130^, which weakly cured [*URE3*] but exhibited no amyloid formation. This anomaly may result from its reduced amyloid-forming ability being dependent on the presence of prion aggregates and undetectable in the SDD-AGE experiment. We also observed that such antiprion activity seems to be a general property of the proteins bearing Q/N-rich domains. The FCS analysis for the molecular detail of prion inhibition dynamics revealed that the curing process involves the enlargement of the [*PSI*^+^] particles of Sup35-GFP in mother cells and subsequently the emergence of huge Sup35-GFP foci.

One may entertain a question as to the mechanism underlying these findings. If Lsm4 and other Q/N-rich proteins possess the bipolar property of both “friends and foes” of yeast prions, how do Lsm4 amyloids bring about a prion loss via the enlargement in the Sup35-GFP amyloids? An important implication in our study is that prion-inducing and prion-curing processes seem to be essentially similar in their underlying mechanisms. Currently, the most widely accepted scenario of [*PSI*^+^] induction is the cross-seeding model (reviewed in Derkatch and Liebman 2007). This model postulates that [*PSI*^+^]-inducible proteins such as Rnq1 first forms amyloid structure, whose growing tip then acts as the template for the amyloid formation of Sup35 proteins. Given the cross-seeding model, the most plausible prion-inhibitory mechanism would be the capping model (Derkatch and Liebman 2007), in which overexpressed Q/N-rich proteins in turn bind to the growing tip of the prion amyloids and inhibit their elongation. However, we have observed that Sup35-GFP amyloid increased, rather than decreasing, its size after the *LSM4* overexpression, which therefore cannot be simply explained by tip–tip interaction models such as the cross-seeding and capping mechanisms.

**Figure 8:**
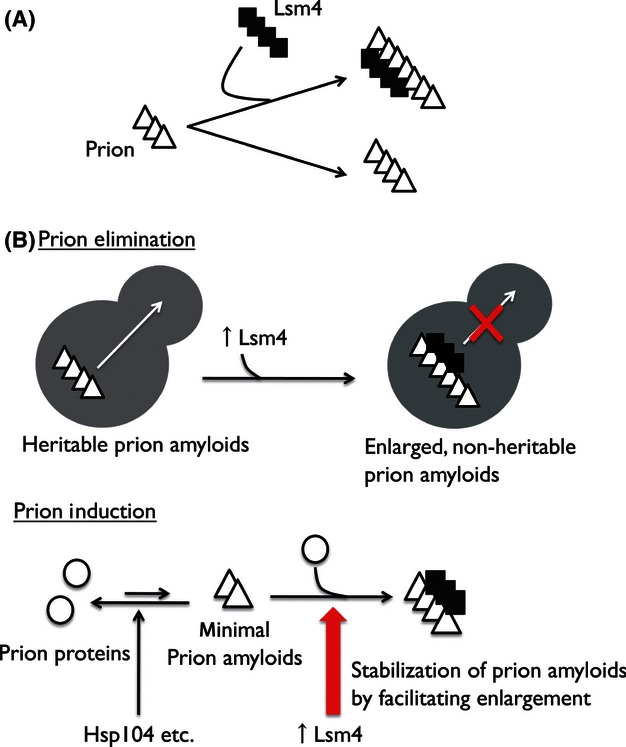
Proposed “unified” mechanism of de novo prion induction and prion elimination. (A) Reaction rate of prion amyloid elongation may be accelerated when the prion is accompanied by Lsm4 amyloid. Black square and white triangle represent Lsm4 and prion proteins, respectively. (B) Assuming that Lsm4 amyloid increases prion particle's size, prion elimination (top) can be interpreted as the size-adding process to thrust large prion amyloids out of the heritable size range to the nonheritable one. Also, de novo prion induction (Pin^+^ effect) (bottom) can be interpreted as the size-adding (and thereby amyloid-stabilizing) process to bring small nascent prion amyloids from lower size limit to the stable one.

Here, we propose the alternative “side-by-side interaction” model. This model predicts that prion amyloid elongation, but not nucleation, is simply facilitated by the side-by-side interaction with heterologous amyloid (diagramed in Fig. 8A). Lansbury's nucleation polymerization (NP) model states that protein oligomerization is a spontaneous but kinetically unfavorable process, while once amyloid nucleus (i.e., amyloid seed) formation is achieved its elongation starts to take place in a kinetically favorable manner (Jarrett and Lansbury 1993). The NP model has been supported by numerous in vitro studies (DePace et al. 1998; Serio et al. 2000; Padrick and Miranker 2002), but we speculate that in cellular environment, de novo amyloid formation is an even-rarer event than the NP model implicates because minimal amyloid seeds are prone to resolubilization owing to the presence of disaggregating chaperone Hsp104 and the mechanical stress by the cytoplasm. Therefore, the catalysis of the elongation step by heterologous amyloids would act to stabilize the prion seeds and, consequently, the prion induction (Fig. 8B). This scenario is consistent to the experimental observation by Lindquist and coworkers that the heterologous prion induction occurred on an amyloid bundle on the IPOD, the cellular compartment for aggregated protein deposition adjacent to the vacuole (Tyedmers et al. 2010). The physical property underlying the elongation catalysis is yet to be investigated, but we speculate that the relative velocity between an amyloid and a monomer, which may negatively influence the kinetics of amyloid elongation, will be lowered when the diffusion coefficient of the amyloid is reduced via a second amyloid accompaniment.

Our model predicts that the facilitation of prion elongation also plays a central role in the prion elimination. Two groups led by Taguchi and Serio recently independently hypothesized that there exists an upper limit of the amyloid size for the proper prion transmission from mother to daughter cells (Derdowski et al. 2010; Taguchi and Kawai-Noma 2010). This implies that the prion amyloids overgrown by *LSM4* overexpression probably surpassed the upper limit of the prion size and led to the prion elimination (Fig. 8B). In fact, several studies have reported that overproduction of prion proteins can disturb their own prion propagation (Edskes et al. 1999; Crapeau et al. 2009; Derdowski et al. 2010), apparently associated with the amyloid enlargement (Crapeau et al. 2009; Derdowski et al. 2010). Our elongation-facilitating model is based on the theory that prion can stably exist only when its amyloid is within the proper size range, thus unifying the mechanism underlying de novo prion induction and elimination as diagramed in Figure 8B. How can the variations of the extent to which [*PSI*^+^] induction and [*URE3*] curing among Q/N-rich proteins be explained (Fig. 7)? Based on our model, the extent should be simply determined by the ability of each Q/N-rich amyloid to elongate the prion amyloid. Possible physical factors that determine the prion regulation strength may include the amyloid-forming propensity of each Q/N-rich protein species and the affinity of each amyloid species to the prion amyloid aggregates.

The FCS data at 120 h after induction showed that the huge foci of Sup35-GFP emerged in the mother cells. Intriguingly, such cell pairs exhibited [*psi*^−^] diffusional profile in the cytoplasm of both daughter and even mother cells. This suggests that the failure of the Sup35-GFP aggregates in the transmission to daughter cells is not simply due to the reduction of diffusion coefficient, rather due to some segregation machinery to recruit prion aggregates toward a compartment of the mother cell. One possible explanation for such aggregate segregation could be that this recruitment is driven by the polarisome-dependent retrograde transport of protein aggregates from daughter to mother cells, which machinery has recently been reported by Nyström and coworkers (Liu et al. 2010). This machinery is believed to be essential for achieving the age asymmetry between mother and daughter cells during the budding stage by the active retention of protein aggregates in mother cells (Liu et al. 2010). Importantly, Hsp104 plays a crucial role in the machinery, recognizing and translocating the protein aggregates to mother cells along the actin cable pathway connected to the polarisome in daughter cells (Liu et al. 2010). Taking this into account, a plausible scenario for the prion propagation failure is that the enlarged, slowly diffusing prion amyloids are more readily trapped by Hsp104 and transported back to the mother cell. Therefore, if the huge foci observed here function as the final destination of trapped prion particles, the foci's identity may be IPOD, a cellular compartment for deposition of irreversibly aggregated proteins such as Rnq1 amyloids (Kaganovich et al. 2008), although the identity remains to be established. Prion propagation would be the event where prion amyloids are small and diffusing fast enough to evade the Hsp104 trap of the protein aggregate segregation machinery.

To sum up, we have discovered that Q/N-rich proteins, including Lsm4, are capable of both upregulating and downregulating prion maintenance in yeast. This study is the generalization of the Q/N-rich protein's bipolar behavior that has repeatedly been glimpsed in our recent studies of numerous Rnq1 mutants. One might ask why Q/N-rich regions are found in diverse kinds of proteins that are usually not involved in the prion biology, and why the regions trigger both the de novo induction and elimination of prions in yeast. Further details need to be investigated, but we speculate that numerous Q/N-rich proteins in the cell, via its own amyloid formation, may play a potential role in the regulation of prion generation and clearance in response to an environmental stress. Nonartificial conditions for the amyloid formation of each Q/N-rich proteins (including Lsm4) are still little known, but various environmental stresses may cause their amyloid formation, via induction of protein expression, defects with protein quality control machinery, and/or oxidative disturbance of their normal conformations.

## Supporting Information

**Figure S1.** Absence of the growth inhibition effect in [*PSI*^+^] or [*URE3*] cells harboring an *LSM4**-*overexpressing plasmid on SC-Leu selective media. (A) Absence of the growth inhibition effect in [*PSI*^+^] cells. [*PSI*^+^][*rnq*^−^] cells (NPK50) were transformed with pRS425 (an empty vector) or pRS425GPDp-LSM4 (a multicopy *LEU2*^+^ plasmid expressing *LSM4* under the control of the *GPD* promoter; denoted by pLSM4). Transformants were subsequently incubated on SC-Leu media for 3 days. (B) Absence of the growth inhibition effect in [*URE3*] cells. [*URE3*][*rnq*^−^] cells (NPK302) were subjected to the same experiment as (A).

**Figure S2.** Cellular abundance and thermotolerance activity of Hsp104 are unaffected by *LSM4* expression. (A) Cellular abundance of Hsp104 on *LSM4* overexpression. *LSM4* was overexpressed from pRS425GPDp-LSM4 (under the *GPD* promoter) in NPK302 [*URE3*][*rnq*^−^] strain. Due to the low amount of lysate loaded, the Lsm4 protein expressed from the genome (see the lane of “Empty vector”) was narrowly observed. Immunoblotting was carried out using anti-Hsp104 antibody, anti-Lsm4 antibody, and anti-Pgk1 antibody. (B) Thermotolerance of *LSM4*-overexpressing cells. Cultures in the mid-log phase were incubated at 37°C for 1 h, and then heat treated at 50°C for 20 min. Survival rates were visualized by fivefold serial dilutions on YPD plate with untreated controls. Used strains were NPK301 ([*ure-o*][*rnq*^−^]) carrying an empty vector (top) or pRS425GPDp-LSM4 (middle), and NPK377 (NPK301-derivative carrying *Δhsp104*; bottom).

**Table S1.** PCR primers.
