# Supplementary material for: A bipolar functionality of Q/N-rich proteins: Lsm4 amyloid causes clearance of yeast prions
Source: Microbiologyopen. 2013 Mar 20;2(3):415–30. doi: 10.1002/mbo3.83 (PMC3684756; doi:10.1002/mbo3.83)
Supplement: Supplementary file 1 [file mbo30002-0415-SD1.pdf]

# SUPPORTING INFORMATION

# A bipolar functionality of Q/N-rich proteins: Lsm4 amyloid causes clearance of yeast prions

Keita Oishi, Hiroshi Kurahashi, Chan-Gi Pack,
Yasushi Sako and Yoshikazu Nakamura*

## Supporting Materials

Table S1. PCR Primers.

| Primer | Sequence (5'-to-3') |
|---|---|
| P1 | GGGGGATCCATGCTACCTTTATATCTTTTAAC |
| P2 | CCCCTCGAGTTAAAATTCGACCTTTTGTG |
| P3 | CCCGAGCTCGATTTCTCGAATAAATC |
| P4 | TTTGTCGACTCCGCTTCCTCCTCCGCTTCCTCCTCCAAATTCGACCTTTTGTGG |
| P5 | CCCGTCGACATGGCCTCCTCCGAGGACG |
| P6 | GGGCTCGAGTTAGGCGCCGGTGGAGTGG |
| P7 | GGGCTCGAGTTACTTGACCTTGTCAATTATATTATC |
| P8 | CCCCTCGAGTTAGTAGTATCTTTTATGCCC |
| P9 | CCCCTCGAGTTAGCCGTTATTATTATTTC |
| P10 | CCCCTCGAGTTAGTTACTGCTGTTGCTGTTG |
| P11 | CCCCTCGAGTTAACCTAAACCATTGTTC |
| P12 | CCCGGATCCATGCAGCAAATTAACTCCAAC |
| P13 | CCCGGATCCAGCGTAATCTGGAACATCGTATGGGTACATACTAGTTCTAGAATCCG |
| P14 | CCCCATATGCTACCTTTATATCTTTTAAC |
| P15 | CCCGGATCCATGCCTCCAAAGAAGTTTAAGG |
| P16 | GGGCTCGAGTCAATCTTCTTCATCGTCAG |
| P17 | CCCGGATCCATGACATCAGTTCAAAAC |
| P18 | GGGCTCGAGTTACATAAGCGTACAACAAAC |
| P19 | CCCGGATCCATGGATTTCTTTAATTTG |
| P20 | GGGCTCGAGTCATTCCAAATTGGTTAGG |

| | |
|---|---|
| P21 | CCCGGATCCATGAACGTTTGCAAGTTG |
| P22 | GGGCTCGAGTTAGTAATATCTATCGCTTTG |
| P23 | CCCGGATCCATGATGAATAACAACGGC |
| P24 | GGGCTCGAGTCATTCACCACGCAATGC |
| P25 | CCCGGATCCATGGAGACCAGTTCTTTTG |
| P26 | GGGCTCGAGTTACCATAGATTCTTCTTG |
| P27 | CCCGGATCCATGAATCCGGGCGGTGAAC |
| P28 | GGGCTCGAGTTAGTCGTCGTAGTTTTC |
| P29 | CCCGGATCCATGATGAATAACAACGGC |
| P30 | GGGCTCGAGTCATTCACCACGCAATGC |
| P31 | CCCAGATCTATGTCTGCTTCATTGATTAATC |
| P32 | GGGCTCGAGTTAAAAGATATTATTAAC |
| P33 | CCCGGATCCATGTCCATGCCCATAGCAAG |
| P34 | GGGCTCGAGTTAGCAACAACCTAATTTTTG |
| P35 | CCCAGATCTATGTTTGGAGTTAGCCGTG |
| P36 | GGGCTCGAGTCAGGTCTGCTCTGCAGCG |

## Supporting Figures

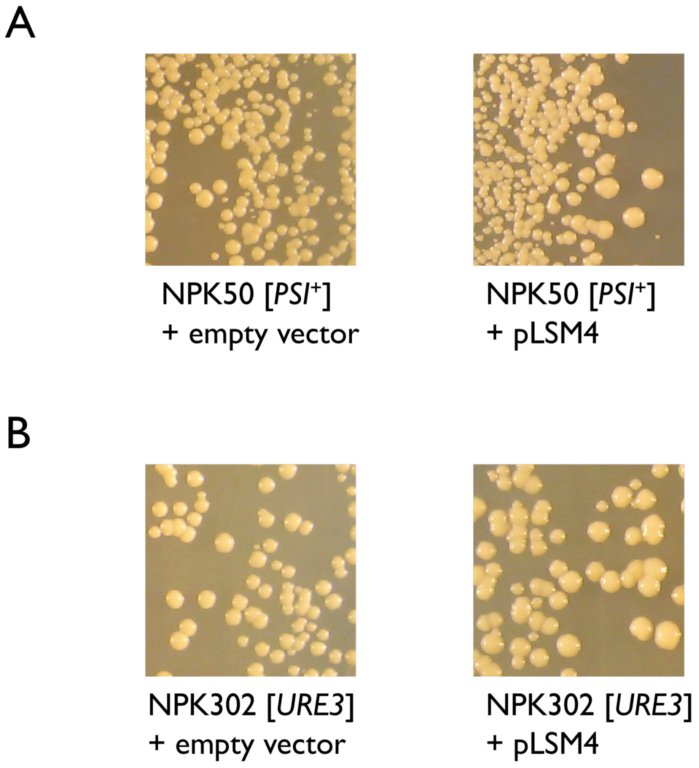


**Fig. S1.** Absence of the growth inhibition effect in [*PSI*$^+$] or [*URE3*] cells harboring an *LSM4* overexpressing plasmid on SC-leu selective media.

A. Absence of the growth inhibition effect in [*PSI*$^+$] cells. [*PSI*$^+$][*rnq*$^-$] cells (NPK50) were transformed with pRS425 (an empty vector) or pRS425GPDp-LSM4 (a multicopy *LEU2*$^+$ plasmid expressing *LSM4* under the control of the *GPD* promoter; denoted by pLSM4). Transformants were subsequently incubated on SC-leu media for 3 days.

B. Absence of the growth inhibition effect in [*URE3*] cells. [*URE3*][*rnq*$^-$] cells (NPK302) were subjected to the same experiment as A.

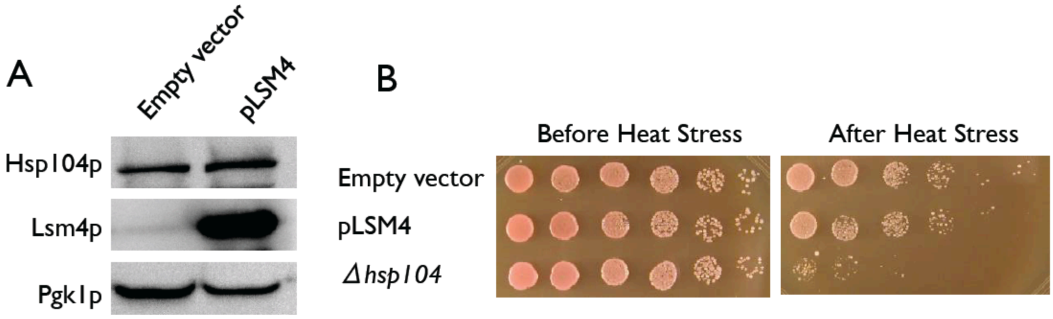


**Fig. S2.** Cellular abundance and thermo-tolerance activity of Hsp104 are unaffected by *LSM4* expression.

A. Cellular abundance of Hsp104 on *LSM4* overexpression. *LSM4* was overexpressed from pRS425GPDp-LSM4 (under the *GPD* promoter) in NPK302 [*URE3*][*rnq*$^-$] strain. Due to the low amount of lysate loaded, the Lsm4 protein expressed from the genome (see the lane of "Empty vector") was narrowly observed. Immunoblotting was carried out using anti-Hsp104 antibody, anti-Lsm4 antibody and anti-Pgk1 antibody.

B. Thermotolerance of *LSM4*-overexpressing cells. Cultures in the mid-log phase were incubated at 37℃ for 1 hour, and then heat-treated at 50℃ for 20 min. Survival rates were visualized by 5-fold serial dilutions on YPD plate with untreated controls. Used strains were NPK301 ([*ure-o*][*rnq*$^-$]) carrying an empty vector (top) or pRS425GPDp-LSM4 (middle), and NPK377 (NPK301-derivative carrying *Δhsp104*; bottom).
